# Detection of autoantibodies to heat shock protein 70 in the saliva and urine of normal individuals

**DOI:** 10.3389/fimmu.2024.1454018

**Published:** 2024-07-29

**Authors:** Krzysztof Sitko, Jagoda Mantej, Marta Bednarek, Stefan Tukaj

**Affiliations:** Department of Molecular Biology, Faculty of Biology, University of Gdańsk, Gdańsk, Poland

**Keywords:** heat shock proteins, Hsps, Hsp70, autoantibodies, saliva, urine, serum, ELISA

## Abstract

Cells exposed to stressors of various origin activate protective mechanisms that include the expression of heat shock proteins (Hsps)/molecular chaperones belonging to several families. Well-characterized inducible Hsp70 is present in all human cell-types and biological fluids, including blood, urine, and saliva. The presence of anti-Hsp70 autoantibodies in the serum of healthy individuals has already been confirmed, and their elevated titers positively correlated with the severity of several pathological conditions, including coeliac disease and dermatitis herpetiformis – a cutaneous manifestation of coeliac disease. Here, using an indirect enzyme-linked immunosorbent assay, we demonstrate, for the first time, that anti-Hsp70 autoantibodies are present in the saliva and urine of healthy individuals. Although the occurrence of anti-Hsp70 autoantibodies in the biological fluids of healthy individuals is intriguing, their physiological role is currently unknown. It is believed that antibodies reacting with self-molecules present in the serum of healthy individuals are part of natural autoantibody pool with multiple regulatory functions. On the other hand, some autoantibodies (e.g., typical of autoimmune bullous skin diseases or systemic lupus erythematosus) may be present before the onset of the disease and serve as specific predictive biomarkers. Therefore, we would like to initiate a discussion or future research direction on the use of anti-Hsp70 autoantibodies as a potential “biomarker” in the diagnosis or prediction of autoimmune diseases. Our findings can be considered in biomedical research to develop noninvasive, inexpensive and easy-to-use tests. Nevertheless, large-scale comparative studies should be initiated, involving the collection and analysis of biological samples such as saliva or urine from patients suffering from autoimmune diseases or other inflammatory or neoplastic diseases, to determine whether the levels of anti-Hsp70 autoantibodies are indeed elevated and whether they correlate with the clinical picture of any disease or established biomarkers.

## 1 Introduction

Autoantibody-driven autoimmune diseases affect more than 2.5% of the population, thus representing a global socio-economic issue (1). The loss of immunological tolerance, a characteristic feature of autoimmune diseases, may lead to the development of self-reacting antibodies (autoantibodies) and, consequently, chronic inflammation and tissue destruction. Autoantibodies can be directed against a variety of intra- and extracellular molecules, such as nucleic acids, lipids, glycoproteins and proteins, becoming a useful tool in the diagnosis and monitoring of some autoimmune diseases (2). In addition, positive result for autoantibodies, in some cases, may be a useful predictive marker. For example, 88% of patients with systemic lupus erythematosus had at least one disease-specific autoantibody present before the disease onset (3). Also, a subset of patients with autoimmune blistering skin diseases had specific autoantibodies directed to various structural skin molecules present from several months up to 10 years before becoming symptomatic (4). On the other hand, it is worth mentioning that a significant portion of healthy individuals have circulating autoantibodies, which may suggest that autoimmunity is not necessarily associated with pathology, and the presence of natural autoantibodies (NAbs) is an important element of the natural immune barrier responsible for protecting the body against bacterial infection or the development of autoimmune diseases (5).

The heat shock response is an evolutionarily conserved mechanism that protects cells from the harmful effects of various stressors, such as heat, cold, toxins, UV radiation, and oxidants. Cells exposed to these stressors activate protective mechanisms, including the expression of heat shock proteins (Hsps). Hsps are a group of constitutive or stress-inducible molecular chaperones that are classified into several families, including Hsp100, Hsp90, Hsp70, Hsp60, Hsp40, and small Hsps. In general, Hsps are responsible for proper folding of newly synthetized polypeptides, refolding of denatured proteins, transport of proteins, and stabilization of the native structure of many polypeptides. The well-characterized 70-kDa stress-inducible heat shock protein (Hsp70) is a key molecular chaperone that is overexpressed in cells exposed to various stressors. Hsp70 can be released (actively or passively) into the extracellular environment under both physiological and stress conditions. Numerous results suggest that both intra- and highly immunogenic extracellular Hsp70 display either pro- or anti-inflammatory activities in the context of development of inflammatory or autoimmune diseases. Such ambiguous roles appear to depend largely on the type of disease. For example, Hsp70-derived epitopes have been found to interact with components of immune cells of the innate and adaptive arms, consequently stimulating a humoral autoimmune response and the production of specific autoreactive antibodies. Anti-Hsp70 autoantibodies have been found to be elevated in patients with various autoimmune diseases, nevertheless, their pathological role and predictive value for the development of autoimmunity are not fully understood (5–7). Although circulating Hsp70 and anti-Hsp70 antibodies have already been found in healthy individuals (8, 9), here, using an indirect enzyme-linked immunosorbent assay, we demonstrate, for the first time, that anti-Hsp70 autoantibodies are also present in the saliva and urine of healthy individuals.

Although no patients were included in this study, we would like to initiate a discussion or future research direction on the use of anti-Hsp70 autoantibodies as a potential “biomarker” in the diagnosis or prediction of autoimmune diseases characterized by an increased titer of anti-Hsp70 autoantibodies in the serum (e.g., rheumatoid arthritis, lupus, dermatitis herpetiformis or coeliac diseases) using biological samples, such as saliva or urine.

## 2 Materials and methods

### Participants

The use of human biological materials was approved by the Bioethics Committee at Regional Medical Chamber in Gdańsk (Poland), and written informed consent was obtained in accordance with the Declaration of Helsinki. The study involved 7 (men, n = 3; women, n = 4; mean age, 30.29; age range, 24 - 42 years) healthy non-smoking volunteers. Over the period of two months, saliva samples (about 2 mL) were collected in the morning using a saliva collection kit (Salivette^®^, Sarstedt). Saliva was separated from the cotton by centrifugation at 1000 RCF for 5 minutes at room temperature (RT) within an hour. The participants were asked to refrain from drinking, eating, and oral hygiene procedures until saliva collection. At the same time, first morning urine (midstream) samples (about 50 mL) were collected directly into a sterile test container. To exclude urinary tract infection, urine was tested using a dipstick method (Reactif Urinalysis, nal von minden GmbH) as per manufacturer’s instructions, and centrifuged at 500 RCF (5 min, RT). In parallel, blood samples (3 mL) were collected, the serum separated by centrifugation. All the collected materials were then stored at -20 ^0^C for further analyses. Participants were not diagnosed with autoimmune diseases and did not report recent viral, fungal or bacterial infections.

### Hsp70

Cloning, expression, and purification of human, stress-inducible Hsp70 (HSPA1A) has been performed as described previously (10). Detailed steps for Hsp70 expression and purification are provided in **Supplementary Data Sheet 1**.

### ELISA

Saliva, urine, and serum levels of immunoglobulins against Hsp70 were evaluated by home-made enzyme-linked immunosorbent assay (ELISA), as described previously with minor modifications (11). Medium-binding 96-well plates were coated with purified endotoxin- and substrate-free human recombinant Hsp70 or bovine serum albumin (BSA; Sigma) at concentrations ranging from 10 to 0.625 μg/mL in 0.1 M bicarbonate buffer at 4°C for 18 hours. The wells were washed with PBS containing 0.05% Tween 20 and blocked with 1% BSA in phosphate-buffered saline (PBS) at RT for 2 h. Following a washing step, saliva, urine, and serum samples (diluted in PBS) were added, and the plates incubated at RT for 90 minutes. After being washed, plates were incubated with horseradish peroxidase (HRP)-conjugated anti-human IgG (Sigma) or IgA (BioLegend) antibodies diluted 1:5000 in PBS containing 0.1% BSA at RT for 60 minutes. TMB substrate solution (Sigma) was used to visualize HRP enzymatic reaction. The reaction was then stopped using 0.5 M H_2_SO_4_. The absorbance was then measured at 450 nm using an ELISA plate reader.

### Statistical analysis

Statistical analyses were performed using the GraphPad Prism 9 software (San Diego, CA). Paired Student’s t-test or Wilcoxon signed-rank test, as well as Pearson’s correlation were used for statistical analysis according to sample distribution as assessed by Shapiro-Wilk’s test. P-values less than 0.05 were considered statistically significant.

## 3 Results and discussion

Well-characterized Hsp70 is expressed in all human cell types and can be found in biological fluids such as serum, urine or saliva (12–14). Although circulating Hsp70 and anti-Hsp70 antibodies have been found in healthy individuals (8, 9), the presence of elevated anti-Hsp70 (auto)antibody titers in the blood has been associated with a plethora of pathological conditions including autoimmune diseases, such as rheumatoid arthritis, dermatitis herpetiformis, coeliac disease, Cogan’s syndrome, myasthenia gravis, Guillain-Barré syndrome, and juvenile idiopathic arthritis (15–17). Nevertheless, their clinical and diagnostic relevancies have not been fully understood and require further investigations. We have previously found that antibodies directed to human Hsp60, Hsp70, and Hsp90 were significantly higher in the serum of patients with dermatitis herpetiformis (Duhring disease) during the active phase of the disease and their levels were significantly lower in remitting patients. Interestingly, levels of anti-Hsp autoantibodies positively correlated with the levels of disease-specific autoantibodies directed against epidermal or tissue transglutaminase (18). Similarly, the titer of circulating autoantibodies against Hsp40, Hsp60, and Hsp90 were significantly higher in patients with coeliac diseases and positively correlated with autoantibodies directed to tissue transglutaminase (19). Another intriguing study showed that patients with systemic lupus erythematosus had at least one disease-specific autoantibody present before the disease onset (3) and a subset of patients with autoimmune blistering skin diseases had specific autoantibodies directed to various structural skin molecules present from several months up to 10 years before becoming symptomatic (4). There are a number of methods, criteria and procedures calling for blood, serum, saliva, or urine sampling, which are commonly used in the diagnosis, prevention and treatment of many diseases. Many medically valuable analytes are gradually being discovered in saliva and urine, some of which are biomarkers of numerous diseases, including cancer and autoimmune diseases. Saliva and urine are important physiological fluids containing a very complex mixture of molecules, including peptides, proteins, glycoproteins, and genetic material. Sampling of such fluids is non-invasive, easy, reproducible and stress-free, requiring minimal sample pre-processing and carrying negligible risk of infection (20). For instance, laboratory blood and urine tests are already used to diagnose and monitor disease activity in patients with systemic lupus erythematosus (21). However, tests that predict the onset or progression of most autoimmune diseases based on monitoring specific and non-invasive biomarkers are not available. The aim of this perspective article is to draw attention to the potential use of non-invasive markers of autoimmune response that may involve anti-Hsp70 autoantibodies present in saliva and urine.

**Figure 1:**
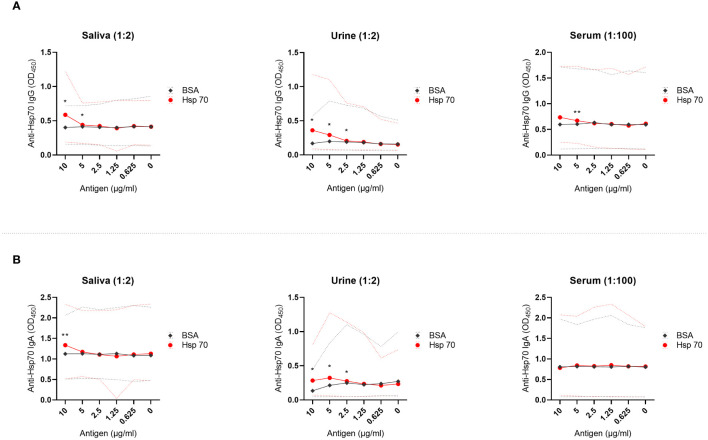
Anti-Hsp70 autoantibodies are present in saliva, urine, and serum of healthy individuals. Saliva, urine, and serum levels of autoantibodies against **(A)** Hsp70 IgG or **(B)** Hsp70 IgA were evaluated by home-made enzyme-linked immunosorbent assay (ELISA), as described previously with minor modifications (11). Optical density measurements were performed at 450 nm (OD_450_) using an ELISA plate reader. OD_450_ values ​​of reactivity of saliva, urine or serum with serially diluted Hsp70 (from 10 to 0 µg/mL) were related to the reactivity to bovine serum albumin (BSA), a negative control. For comparative analyses, depending on the distribution of variables, the Student’s t-test or the Wilcoxon signed-rank test was used. P-value less than 0.05 was considered statistically significant. The dilution of saliva, urine, or serum in PBS is given above the graph. The results are presented as solid lines of mean values of 7 healthy individuals. Dashed lines represent min-max values. *P < 0.05, **P < 0.01. Raw data are presented in **Supplementary Data 2**.

While the reactivity of saliva, urine, and serum to increasing concentrations of plate-bound bovine serum albumin (BSA; control protein) was unchanged, dose-dependent reactivity of these biological fluids to Hsp70 was observed (**Figure 1**). Specifically, we observed the presence of IgG autoantibodies against Hsp70 in saliva, urine, and serum (**Figure 1A**). Of note, levels of anti-Hsp70 IgG present in saliva positively correlated with the levels of anti-Hsp70 IgG present in urine (Pearson’s correlation; R = 0.775, p-value = 0.041). We then recorded the presence of anti-Hsp70 IgA antibodies in saliva and urine, with no change in the serum levels of these antibodies (**Figure 1B**).

Although the occurrence of anti-Hsp70 autoantibodies in the biological fluids such as serum, saliva and urine of healthy individuals is intriguing, their physiological role is currently unknown. We would like to open a discussion on using anti-Hsp70 autoantibodies of various isotypes as a potential “biomarker” in diagnosis or prediction of autoimmune disorders characterized by an increased titer of anti-Hsp70 autoantibodies in the serum. We believe that our findings might be considered in biomedical studies for the development of non-invasive, inexpensive, and easy-to-use tests. Therefore, extensive comparative research studies involving the collection and analysis of biological samples, such as saliva or urine from patients suffering from autoimmune disorders need to be initiated to determine whether anti-Hsp70 autoantibody levels are indeed elevated and whether they correlate with the clinical picture of any disease or previously established biomarkers. Nevertheless, restrictive rules on sampling and storage conditions for biological material must be followed, as well as appropriate cut-off values established, in the further comparative analyses.

## 4 Limitations

It is important to acknowledge the limitations of this preliminary observation. Critically, no patients were included in the study which means the hypothetical usefulness of measuring reactivity against self-Hsps as biomarkers at this moment is speculative. We are also aware that the small number of healthy individuals included in this study may not be representative of larger populations. Therefore, future sampling should include a larger number of healthy donors and patients and consider the ethnic diversity, genetic background, and gut microbiome of patients (22, 23). Finally, future studies should also determine whether the presence of autoantibodies directed against Hsp70 in saliva and urine constitute NAbs. These autoantibodies, consisting of mainly IgM, IgA (IgA1 and IgA2) and IgG (IgG1, IgG2, IgG3, and IgG4), bind to exogenous antigens (e.g., bacterial) acting as a first line of immune defense against infections and can be polyreactive (24). NAbs also bind to and remove neo-autoantigens that are revealed during cell or tissue damage. They have also been described as protectors from autoimmunity (25, 26). Although there are several scientific reports indicating the occurrence of NAbs targeting Hsps in the blood (27–32), their protective or pathogenic role requires further investigations (5). Therefore, we believe that future studies – inspired by the preliminary observations presented in this paper and other studies on this topic – will provide new knowledge regarding the defensive or pathogenic mechanisms resulting from the presence of autoantibodies directed against autologous heat shock proteins.

## Data availability statement

The original contributions presented in the study are included in the article/**Supplementary Material**. Further inquiries can be directed to the corresponding author.

## Ethics statement

The studies involving humans were approved by Bioethics Committee at Regional Medical Chamber in Gdańsk (Poland). The studies were conducted in accordance with the local legislation and institutional requirements. The participants provided their written informed consent to participate in this study.

## Author contributions

KS: Data curation, Formal Analysis, Investigation, Methodology, Software, Validation, Visualization, Writing – review & editing. JM: Formal Analysis, Investigation, Writing – review & editing. MB: Data curation, Formal Analysis, Investigation, Methodology, Writing – review & editing. ST: Conceptualization, Data curation, Funding acquisition, Investigation, Methodology, Project administration, Supervision, Writing – original draft, Writing – review & editing.
