# Supplementary material for: Detection of autoantibodies to heat shock protein 70 in the saliva and urine of normal individuals
Source: Front Immunol. 2024 Jul 29;15:1454018. doi: 10.3389/fimmu.2024.1454018 (PMC11317234; doi:10.3389/fimmu.2024.1454018)
Supplement: Supplementary file 1 [file DataSheet_1.pdf]

Supplementary data 1.

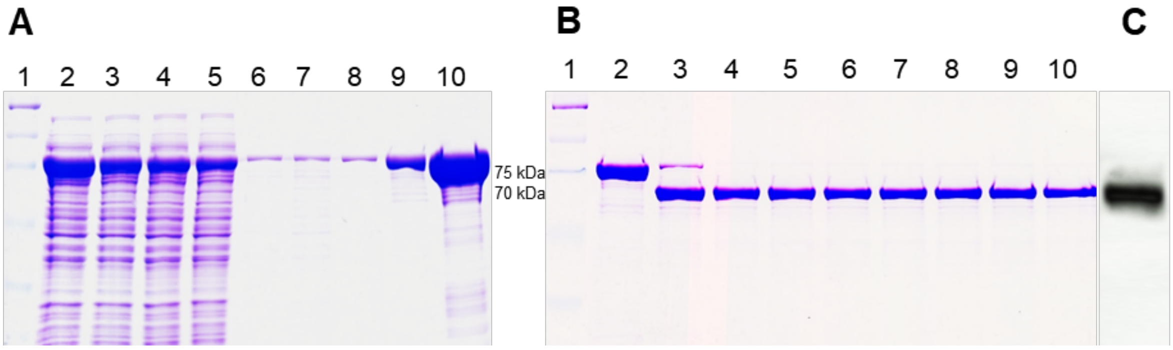

Cloning, Expression, and Purification of Hsp70. Synthetic DNA fragment encoding Hsp70 (HSPA1A) from *Homo sapiens* (NP_005336.3) has been obtained from Thermo Scientific (GeneArt service). Codon usage was optimized for efficient gene expression in *E. coli* by the GeneOptimizer software. The insert was synthesized with N-terminal 6x-His-SUMO tag and cloned into the pET151/TOPO (Thermo Scientific) plasmid. Lipopolysaccharide-free E. coli BL21 (DE3) ClearColi (Lucigen) strain carrying the plasmid was grown in the LB medium supplemented with 1% NaCl, 1 mM IPTG (Sigma), and ampicillin at 18 °C, overnight. The use of ClearColi cells ensured that the purified protein was free from lipopolysaccharide contamination. Cells were harvested by centrifugation, resuspended in a lysis buffer (20 mM Tris-HCl pH 8.0, 500 mM NaCl, 20 mM imidazole, 10% glycerol, ROCHE protease inhibitor cocktail), and disrupted by sonication. After centrifugation, the supernatant was loaded on the HIS-Select® Nickel Affinity Gel resin (Sigma) equilibrated with the lysis buffer. To remove unbound proteins and the chaperone-associated substrates, the column was washed with a buffer containing 5 mM ATP, 5 mM $MgCl_2$, 1 M NaCl, and 20 mM Tris-HCl pH = 8.0. The Hsp70 containing fractions (eluted with lysis buffer containing 180 mM imidazole) were dialyzed against a dialysis buffer (20 mM Tris-HCl pH = 8.0, 250 mM NaCl, 10% glycerol), followed by His-tag cleavage using SUMO protease (Sigma). To remove His-tag from the mixture, the protein sample was loaded on the HIS-Select® Nickel Affinity Gel resin (Sigma) equilibrated with the dialysis buffer. The Hsp70 fraction (99% purity) was filtered (0.22 µm) and stored at − 80 °C.

10% Coomassie Brilliant Blue stained SDS-PAGE gel showing Hsp70 purification steps.

Supplementary data 1A. Lane 1 – molecular weight marker; line 2 - bacterial lysate obtained by sonication; lane 3 and 4 - fractions collected after incubation of the lysate in HIS-Select® Nickel Affinity Gel; line 5 and 6 - fractions collected after washing the bed with lysis buffer; lane 7 and 8 - fractions collected after washing the bed with a buffer containing ATP; lanes 9 and 10 - fractions containing Hsp70 fused to His-SUMO tag.

Supplementary data 1B. Lane 1 – molecular weight marker; lane 2 - Hsp70 fused to His-SUMO tag; lane 3 - fraction collected after digestion with the SUMO enzyme; lane 4-10 - fractions collected after washing HIS-Select® Nickel Affinity Gel with imidazole buffer, containing pure Hsp70 protein, without His tag.

Immunoblotting

Supplementary data 1C. Purified Hsp70 was separated on a 10% polyacrylamide gel under denaturing conditions. Western blots were performed using monoclonal mouse anti-Hsp70 IgG1 antibodies (clone BRM-22; Sigma) as primary antibodies and HRP-conjugated goat anti-mouse immunoglobulins as secondary antibodies. Detection was made on an X-ray film using a substrate for HRP (Western Blotting Amersham ECL Plus).
