# Supplementary material for: Detection of autoantibodies to heat shock protein 70 in the saliva and urine of normal individuals
Source: Front Immunol. 2024 Jul 29;15:1454018. doi: 10.3389/fimmu.2024.1454018 (PMC11317234; doi:10.3389/fimmu.2024.1454018)
Supplement: Supplementary file 2 [file DataSheet_2.pdf]

Subject / Antigen (µg/ml)	1	2	3	4	5	6	7	1	2	3	4	5	6	7
**Urine 1:2**														
**Group**	**Anti-Hsp70 IgG**							**BSA**						
10	0.213	0.225	0.138	1.179	0.455	0.091	0.219	0.102	0.102	0.086	0.569	0.148	0.072	0.097
5	0.144	0.168	0.098	1.103	0.292	0.078	0.164	0.097	0.094	0.083	0.79	0.153	0.070	0.101
2.5	0.109	0.110	0.084	0.761	0.189	0.072	0.118	0.097	0.091	0.077	0.728	0.163	0.070	0.103
1.25	0.101	0.095	0.080	0.706	0.160	0.073	0.104	0.096	0.088	0.079	0.684	0.139	0.067	0.112
0.625	0.096	0.098	0.087	0.521	0.140	0.070	0.100	0.098	0.085	0.077	0.566	0.135	0.068	0.100
0	0.097	0.087	0.078	0.464	0.163	0.066	0.108	0.095	0.092	0.075	0.511	0.159	0.071	0.100
**Group**	**Anti-Hsp70 IgA**							**BSA**						
10	0.211	0.158	0.105	0.806	0.409	0.073	0.225	0.124	0.051	0.053	0.445	0.162	0.047	0.059
5	0.210	0.109	0.081	1.276	0.383	0.059	0.148	0.202	0.057	0.063	0.837	0.234	0.048	0.063
2.5	0.195	0.075	0.080	1.138	0.304	0.053	0.083	0.180	0.060	0.073	1.100	0.219	0.052	0.063
1.25	0.184	0.063	0.079	0.983	0.215	0.054	0.068	0.166	0.068	0.076	0.960	0.189	0.051	0.063
0.625	0.143	0.062	0.076	0.613	0.427	0.080	0.095	0.174	0.059	0.082	0.781	0.414	0.065	0.089
0	0.192	0.058	0.078	0.735	0.408	0.078	0.089	0.162	0.060	0.074	0.996	0.456	0.072	0.085
**Saliva 1:2**														
**Group**	**Anti-Hsp70 IgG**							**BSA**						
10	0.304	0.229	0.833	1.219	0.973	0.189	0.362	0.255	0.206	0.600	0.719	0.594	0.155	0.286
5	0.259	0.219	0.631	0.751	0.758	0.171	0.287	0.246	0.214	0.596	0.721	0.692	0.157	0.267
2.5	0.246	0.200	0.619	0.774	0.682	0.151	0.286	0.238	0.204	0.609	0.743	0.621	0.144	0.281
1.25	0.248	0.058	0.606	0.797	0.616	0.143	0.277	0.213	0.197	0.611	0.801	0.567	0.137	0.280
0.625	0.231	0.200	0.597	0.790	0.688	0.149	0.302	0.230	0.195	0.573	0.821	0.665	0.140	0.286
0	0.225	0.192	0.583	0.795	0.653	0.144	0.303	0.225	0.190	0.557	0.859	0.622	0.130	0.310
**Group**	**Anti-Hsp70 IgA**							**BSA**						
10	1.156	0.514	1.861	1.056	2.333	0.832	1.608	0.810	0.509	1.513	0.739	2.061	0.68	1.550
5	0.950	0.571	1.419	0.754	2.181	0.792	1.535	0.788	0.522	1.439	0.738	2.269	0.715	1.428
2.5	0.819	0.504	1.307	0.755	2.172	0.758	1.439	0.791	0.519	1.365	0.76	2.199	0.693	1.460
1.25	0.831	0.041	1.448	0.768	2.202	0.701	1.466	0.838	0.495	1.386	0.779	2.256	0.670	1.489
0.625	0.763	0.495	1.279	0.752	2.305	0.729	1.445	0.649	0.454	1.308	0.770	2.304	0.668	1.427
0	0.097	0.087	0.078	0.464	0.163	0.066	0.108	0.095	0.092	0.075	0.511	0.159	0.071	0.100
**Serum 1:100**														
**Group**	**Anti-Hsp70 IgG**							**BSA**						
10	1.723	0.399	0.75	0.471	1.108	0.249	0.439	1.716	0.121	0.799	0.119	0.826	0.181	0.405
5	1.727	0.231	0.833	0.258	1.001	0.237	0.405	1.676	0.128	0.795	0.122	0.899	0.212	0.384
2.5	1.656	0.160	0.835	0.157	0.932	0.222	0.371	1.669	0.126	0.918	0.172	0.928	0.207	0.403
1.25	1.689	0.135	0.808	0.131	0.861	0.209	0.398	1.564	0.131	0.828	0.130	0.890	0.200	0.414
0.625	1.571	0.132	0.723	0.118	0.879	0.215	0.381	1.640	0.124	0.828	0.122	0.848	0.199	0.409
0	1.718	0.125	0.845	0.112	0.878	0.204	0.392	1.604	0.128	0.778	0.119	0.900	0.198	0.422
**Group**	**Anti-Hsp70 IgA**							**BSA**						
10	1.267	0.108	0.662	0.237	0.983	0.135	2.077	1.168	0.075	0.872	0.164	1.287	0.117	1.973
5	1.276	0.094	0.827	0.246	1.281	0.139	2.041	1.215	0.081	0.833	0.200	1.419	0.125	1.839
2.5	1.124	0.082	0.760	0.192	1.227	0.129	2.261	1.139	0.091	0.770	0.187	1.380	0.127	1.969
1.25	1.201	0.087	0.764	0.178	1.253	0.122	2.336	1.157	0.078	0.815	0.181	1.231	0.119	2.062
0.625	1.132	0.079	0.767	0.169	1.409	0.120	2.068	1.173	0.075	0.839	0.162	1.513	0.117	1.838
0	1.188	0.076	0.799	0.175	1.546	0.131	1.790	1.127	0.075	0.811	0.182	1.539	0.127	1.761
